# Preparation of Steamed Purple Sweet Potato-Based Films Containing Mandarin Essential Oil for Smart Packaging

**DOI:** 10.3390/molecules29102314

**Published:** 2024-05-15

**Authors:** Ruixue Yue, Yiren Zhang, Jun Liu, Jian Sun

**Affiliations:** 1 Xuzhou Institute of Agricultural Sciences, Jiangsu Xuhuai Area, Xuzhou 221131, China; yueruixue_1983@163.com; 2 College of Food Science and Engineering, Yangzhou University, Yangzhou 225127, China; 212403120@stu.yzu.edu.cn

**Keywords:** freshness tracing, mandarin essential oil, purple sweet potato, smart packaging

## Abstract

Anthocyanin-rich steamed purple sweet potato (SPSP) is a suitable raw material to produce smart packaging films. However, the application of SPSP-based films is restricted by the low antimicrobial activity of anthocyanins. In this study, SPSP-based smart packaging films were produced by adding mandarin essential oil (MEO) as an antimicrobial agent. The impact of MEO content (3%, 6%, and 9%) on the structures, properties, and application of SPSP-based films was measured. The results showed that MEO created several pores within films and reduced the hydrogen bonding system and crystallinity of films. The dark purple color of the SPSP films was almost unchanged by MEO. MEO significantly decreased the light transmittance, water vapor permeability, and tensile strength of the films, but remarkably increased the oxygen permeability, thermal stability, and antioxidant and antimicrobial properties of the films. The SPSP-MEO films showed intuitive color changes at different acid-base conditions. The purple-colored SPSP-MEO films turned blue when chilled shrimp and pork were not fresh. The MEO content greatly influenced the structures, physical properties, and antioxidant and antimicrobial activities of the films. However, the MEO content had no impact on the color change ability of the films. The results suggested that SPSP-MEO films have potential in the smart packaging of protein-rich foods.

## 1. Introduction

Traditional plastic-based packaging films have been utilized to protect food products for several decades. However, due to their non-degradability, traditional plastic-based packaging is insufficient to meet the standard of modern food industry and circular economy [1]. Smart packaging, a green and sustainable food packaging technology, has been increasingly exploited in the past ten years [2]. Smart packaging can be subdivided into active packaging and intelligent packaging [3]. Active packaging aims to maintain the quality of food products through releasing active components (e.g., antimicrobials and antioxidants) into the preserved food or absorbing metabolites (e.g., carbon dioxide, oxygen, and ethylene) from food products [4]. Intelligent packaging aims to monitor the quality of food products by using indicators, chemical sensors, and biosensors to trace the physiological status (e.g., pH level and freshness degree) and environmental conditions (e.g., temperature, humidity, and atmosphere) of food products during transportation and storage [5]. Therefore, smart packaging techniques play important roles in the food packaging sector.

In recent years, plant anthocyanins have normally been incorporated into biodegradable polymers to fabricate smart packaging films [6,7]. Anthocyanins, due to their polyphenolic nature, are potent antioxidants that can inhibit food oxidation [8]. Anthocyanins, as color variable substances, can be used to trace the freshness degree of food products by sensing the atmosphere changes during food spoilage [9]. However, the application of anthocyanin-based smart packaging films is restricted by the limited antimicrobial activity of anthocyanins. In order to improve the antimicrobial activity of anthocyanin-based smart packaging films, several researchers managed to add antimicrobials such as essential oils [10], quantum dots [11], and metal nanoparticles [12] into the films. Among these antimicrobials, essential oils are of natural origin and are more suitable for food packaging [13]. The antimicrobial activity of essential oils is attributed to the presence of plant secondary metabolites, such as terpenes, phenylpropanoids, aldehydes, esters, alcohols, and ketones [14]. These secondary metabolites exhibit antimicrobial potential through damaging cytoplasmic membrane, alternating the fatty acid profile of cell membranes, and reducing proton-motive force [15]. Essential oils isolated from different plants including molle [16], oregano [10,17,18], curcuma [19], lemongrass [20], neem [21], chamomile [22], cinnamon [23,24], thyme [25,26] and rosemary [27] have previously been used to increase the antimicrobial activity of anthocyanins-based packaging films.

Purple sweet potato is a global crop with purple flesh, which is attributed to the presence of anthocyanins [28]. Several studies have previously reported the preparation of smart packaging films using purple sweet potato extract containing anthocyanins [10,28,29,30]. However, the preparation of smart packaging films by simultaneously using purple sweet potato anthocyanins and plant essential oils has seldom been considered by researchers [10]. Notably, except for anthocyanins, other ingredients (e.g., starch and soluble and insoluble dietary fibers) are also included in purple sweet potato, which can serve as the matrix of packaging films. A recent study has highlighted that smart packaging films can easily be prepared by using steamed purple sweet potato (SPSP), which not only saves on the extraction cost of anthocyanins but also increases their stability [31]. Although SPSP-based packaging films have good intelligent packaging potential to trace the freshness degree of food products, the films possess low antioxidant and antimicrobial activities.

Mandarin (*Citrus reticulata*) is one of the major cultivated citrus fruits around the world [32]. The peels of citrus fruits are rich in essential oils with antioxidant and antimicrobial activities [33]. In recent years, many researchers have managed to incorporate citrus essential oils (e.g., orange, mandarin, bergamot, lemon, and lime essential oils) into food packaging films [34,35]. The incorporation of citrus essential oils normally decreases the water affinity, transparency, and tensile strength of the films but increases their elongation at break and antioxidant and antimicrobial activities [34,35]. However, only a few studies have previously focused on preparing food packaging films using mandarin essential oil (MEO) [36,37,38]. Therefore, it is supposed that MEO can be added into SPSP-based packaging films to increase the antioxidant and antimicrobial activities of the films.

In the present study, smart packaging films were prepared by adding MEO into an SPSP-based matrix. This study aimed to investigate the impact of the MEO content (3%, 6%, and 9%) on the structural characteristics, physical, and functional properties of the films. The ability of the smart packaging films to trace the freshness of shrimp and pork was also tested.

## 2. Results and Discussion

### 2.1. Structural Characterization of Films

#### 2.1.1. SEM Images

**Figure 1:**
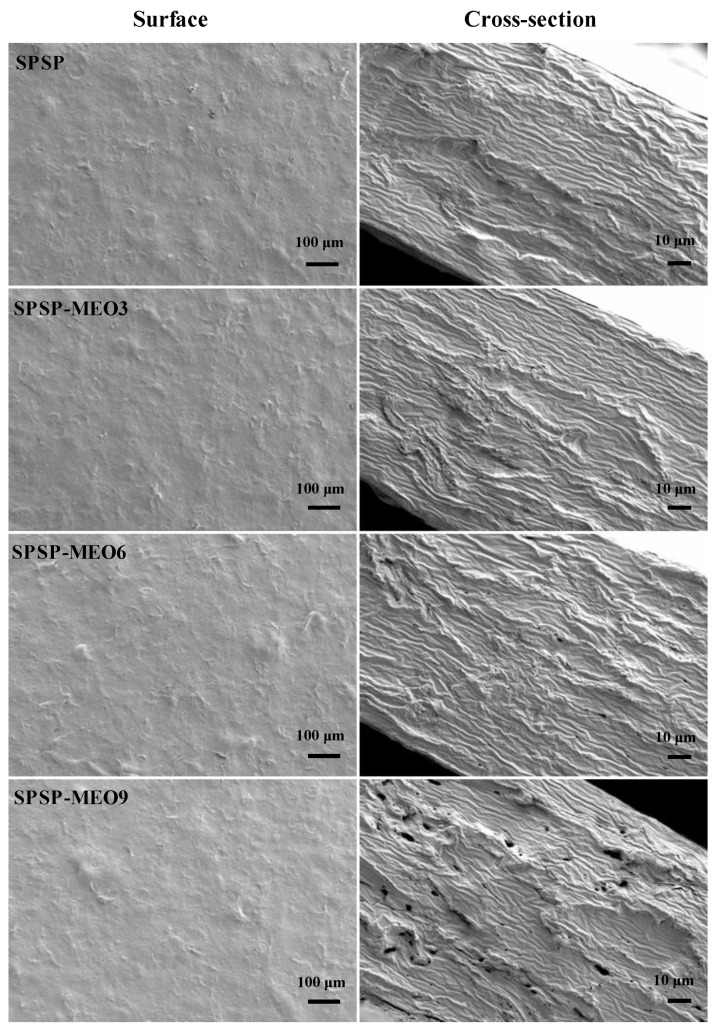
Surface and cross-section morphologies of SPSP film, SPSP-MEO3 film, SPSP-MEO6 film, and SPSP-MEO9 film.

The surface and cross-section of the SPSP film, SPSP-MEO3 film, SPSP-MEO6 film, and SPSP-MEO9 film are shown in Figure 1. All films exhibited similar surface morphologies, with some humps randomly distributed on film surfaces. The humps on film surfaces were probably caused by the insoluble dietary fibers in purple sweet potato that had low compatibility with other film components (e.g., sodium alginate, starch, and soluble dietary fibers in purple sweet potato). The SPSP film had a compact cross-section without cracks or pores, indicating that film components closely interacted with each other. Some protrusions were noted in the cross-section of the SPSP film, which were caused by the tangled fibers. Different from the SPSP film, the SPSP-MEO3 film, SPSP-MEO6 film, and SPSP-MEO9 film showed several pores in their cross-sections. In addition, the number and size of pores of the SPSP-MEO films increased with the content of MEO. The void cavities within the films were caused by hydrophobic MEO that could aggregate and volatilize during film formation. Higher contents of MEO had more opportunities to aggregate into larger oil droplets and to volatilize into bigger cavities. Several other researchers also found that the films with essential oils had several inner void cavities [10,17,20,24,26]. The above results suggested that the cross-section of the films was greatly affected by the content of MEO.

#### 2.1.2. FT-IR Spectra

**Figure 2:**
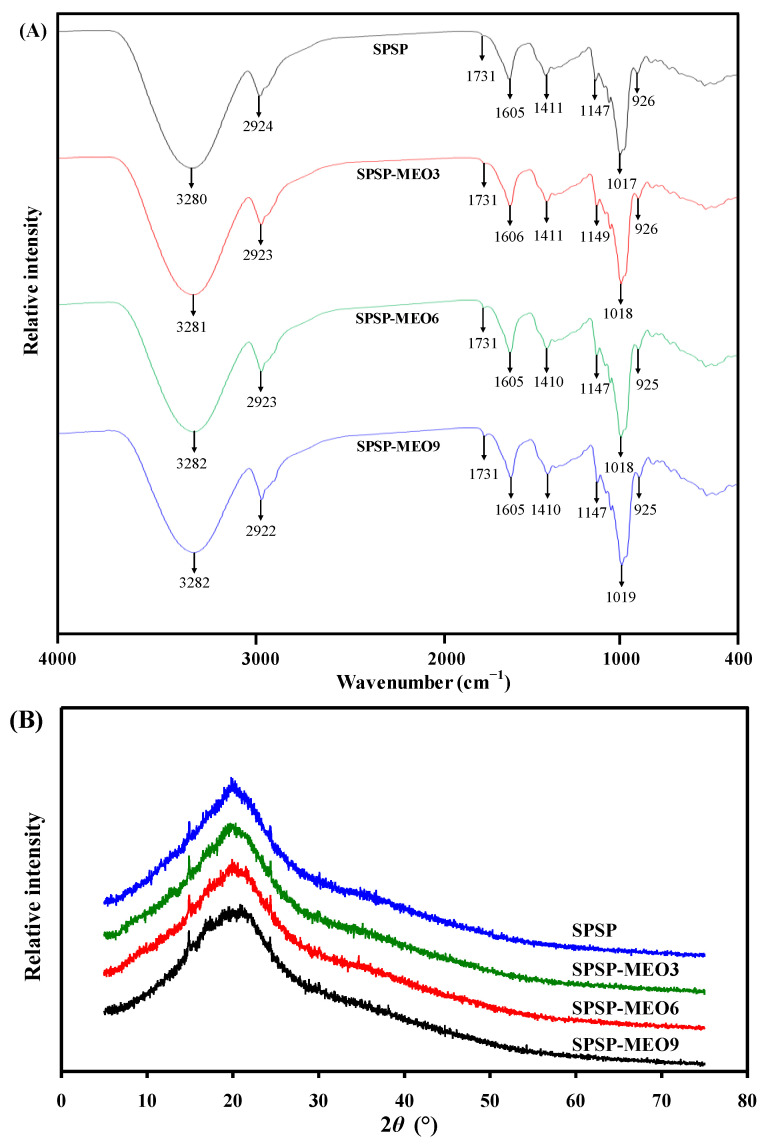
FT-IR spectra (**A**) and XRD patterns (**B**) of SPSP film, SPSP-MEO3 film, SPSP-MEO6 film, and SPSP-MEO9 film.

The FT-IR spectra of the SPSP film, SPSP-MEO3 film, SPSP-MEO6 film, and SPSP-MEO9 film are shown in Figure 2A. the SPSP film exhibited IR bands at 3280, 2924, 1731, 1605, 1411, and 1017–1147 cm^−1^, attributed to –OH/–NH stretching, –CH_2_ stretching, C=O stretching, C=C stretching, CH–CH_2_ bending, and C–O–C stretching, respectively [18,26]. These IR bands were caused by multiple components in purple sweet potato, such as starch and insoluble and soluble dietary fibers. Meanwhile, other film components including sodium alginate and glycerol also contributed to the IR bands of the SPSP film [31]. The position of IR bands was not remarkably changed by MEO. However, the amplitude of IR bands at 3280 and 1731 cm^−1^ were significantly altered after adding MEO. With the increase in MEO content, the band at 3280 cm^−1^ gradually decreased whereas the band at 1731 cm^−1^ gradually increased. The decreased band at 3280 cm^−1^ was caused by MEO that disrupted the hydrogen bonding system in the SPSP film. Similar results were observed in other films containing anthocyanins and essential oils [16,20]. The increased band at 1731 cm^−1^ was attributed to the ester groups in MEO [24]. The above results indicated that the content of MEO negatively affected the hydrogen bonding interactions of film components.

#### 2.1.3. XRD Patterns

As displayed in Figure 2B, the XRD figures of the SPSP film, SPSP-MEO3 film, SPSP-MEO6 film, and SPSP-MEO9 film are very similar. The SPSP film presented three crystalline peaks at 15.0° (cellulose I), 20.2° (cellulose II), and 24.4° (cellulose I), corresponding to the diffraction of cellulose [39]. The SPSP-MEO films had similar cellulose I crystalline structures to those of the SPSP film. A similar phenomenon was observed by Chen et al. [10], who found that the crystalline structure of cellulose I was unaffected by oregano essential oil. However, the crystalline structure of cellulose II was significantly changed by MEO. With the increase in MEO content, the crystalline peak of cellulose II tended to become amorphous. This indicated that the original crystalline structure of the SPSP film was partially disrupted by MEO, which was in line with the declined hydrogen bonding system in the SPSP-MEO films (Figure 2A). The above results suggested that the crystalline structures of the films were influenced by the content of MEO.

### 2.2. Physical Properties of Films

#### 2.2.1. Color

As shown in Table 1, all films were transparent and dark purple without significant thickness differences (*p* > 0.05). Notably, the dark purple color of the films was the same as the color of the raw material (SPSP). This was because the pH value of the film-forming solutions was 6.4–6.5, which had no influence on the color of the anthocyanins in SPSP. With the increase in MEO content, the three SPSP-MEO films showed decreased a* values (redness ↓) but increased b* values (yellowness ↑). This was because MEO is a light yellow substance. As a consequence, the three SPSP-MEO films exhibited decreased Δ*E* values. Despite this, none of the films showed obvious, naked-eye-distinguishable color differences. Therefore, the content of MEO had little impact on the color of the SPSP films. Likewise, other researchers found that the color of films containing anthocyanins was almost unchanged by essential oils [18,40].

#### 2.2.2. Barrier Properties

**Figure 3:**
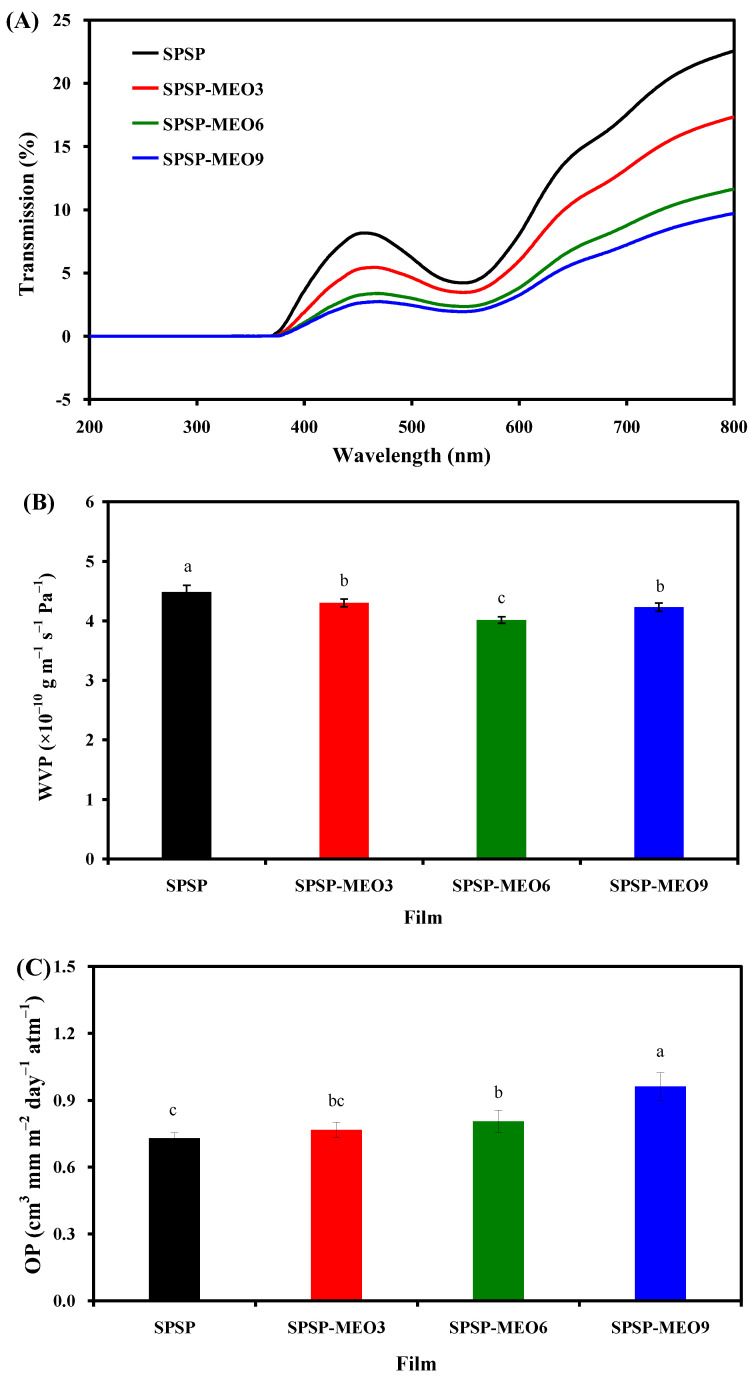
Barrier properties of SPSP film, SPSP-MEO3 film, SPSP-MEO6 film, and SPSP-MEO9 film against UV-vis light (**A**), water vapor (**B**), and oxygen gas (**C**). Data are given as mean ± SD (*n* = 3). Data with different lower case letters indicates significantly different values (*p* < 0.05).

Food products are sensitive to light radiation, and smart packaging films should be able to block UV–vis light. The UV–vis transmittance of the SPSP film, SPSP-MEO3 film, SPSP-MEO6 film, and SPSP-MEO9 film was tested at 200–800 nm (Figure 3A), where all films had zero light transmission at wavelengths smaller than 376 nm. When the wavelength was greater than 376 nm, the light transmittance of the films decreased with the increase in MEO content. All films displayed a significant transmittance reduction at 540 nm, which was assigned to the absorption of anthocyanins [24]. The above results indicated that the content of MEO had a positive impact on the light barrier ability of the films, which was also demonstrated by other researchers [16,19,24,27]. This was because plant essential oils have several chromogenic groups, such as aromatic rings [10]. In addition, the lipid droplets distributed in the films could refract and scatter light, which further contributed to the light barrier ability of the films [16]. SPSP-MEO films with potent light barrier ability were able to protect light-sensitive foods.

WVP is often used to indicate the capacity of smart packaging films to prevent moisture transfer between the atmosphere and foods. As displayed in Figure 3B, the SPSP film had a higher WVP than the three SPSP-MEO films (*p* < 0.05). The high WVP of the SPSP film was attributed to the hydrophilic substances in the film, such as starch, soluble dietary fiber, sodium alginate, and glycerol. The WVP of the SPSP film was lowered by MEO, which was because MEO is a hydrophobic substance with water-proofing properties [18,20]. Meanwhile, MEO disrupted the hydrogen bonding system of the films, as demonstrated by the reduced –OH stretching in the SPSP-MEO films (Figure 2A). Other researchers also found that the WVP of the films containing anthocyanins was lowered by essential oils [18,20,21]. It is worth noting that the WVP of the SPSP-MEO films was influenced by the MEO content. With the increase in MEO content, the WVP of the SPSP-MEO films gradually decreased and then increased (*p* < 0.05). This was because the WVP of the films was not only influenced by hydrophobic essential oils but also affected by the microstructure of the films [23]. As presented in Figure 1, the porous structure of the SPSP-MEO films became more and more obvious when the MEO content increased, which increased the route of water vapor. Zhang et al. [18] and Zhao et al. [24] also demonstrated that the lowest WVP was achieved when a certain amount of plant essential oils was added into films containing anthocyanins. In the present study, the results indicated that the SPSP-MEO films could effectively delay water vapor transfer between the atmosphere and foods.

Oxygen is one of the causes of food deterioration, and thus smart packaging films should have good oxygen barrier capacity. As displayed in Figure 3C, the three SPSP-MEO films had a higher OP than the SPSP film (*p* < 0.05). Meanwhile, the OP of the SPSP-MEO films progressively increased when the MEO content increased (*p* < 0.05). This was because the SPSP-MEO films contained several inner cavities that supplied passages for oxygen gas (Figure 1). Since the SPSP-MEO 9 film had the biggest cavities, it presented the highest OP (*p* < 0.05). By contrast, the SPSP film had a relatively lower OP (*p* < 0.05) because its inner microstructure was compact. Therefore, the inner microstructure of the SPSP and SPSP-MEO films greatly affected the OP of the films. Only a few researchers have previously tested the OP of films containing anthocyanins and essential oils [20,21]. Li et al. [20] concluded that the OP of the films was increased by lemongrass essential oil, which was because the essential oil created porous microstructure within the films. However, Yang et al. [21] did not observe obvious OP changes after neem essential oil was added to the films, which might be related to the unchanged microstructure of the films. In the present study, the results suggested that the content of MEO had a negative impact on the oxygen barrier capacity of the films. Despite this, the SPSP-MEO films still possessed a certain oxygen barrier capacity that could protect foods against oxidation.

#### 2.2.3. Mechanical Properties

**Figure 4:**
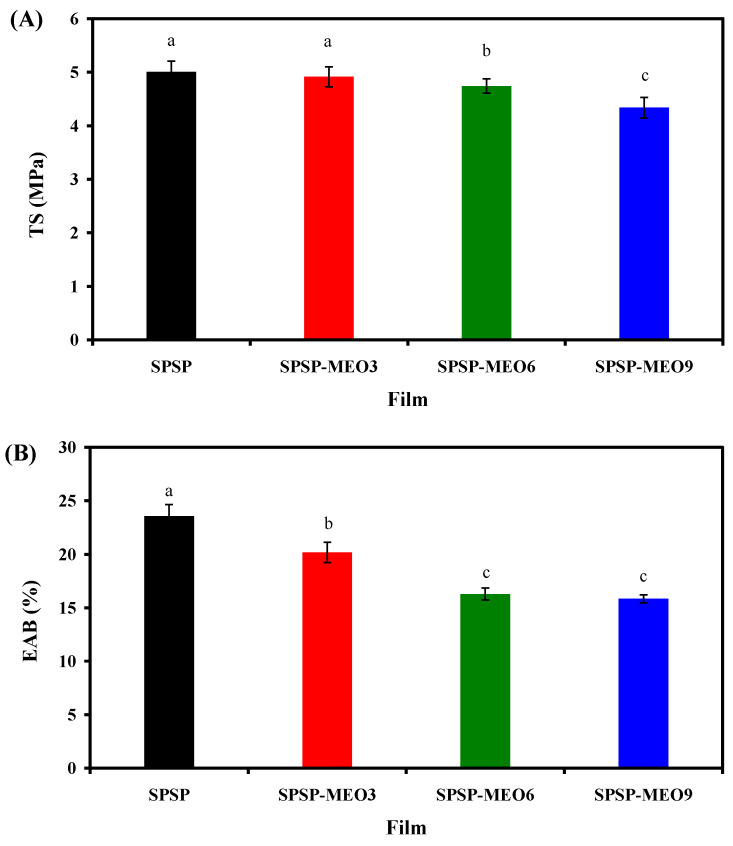
TS (**A**) and EAB (**B**) of SPSP film, SPSP-MEO3 film, SPSP-MEO6 film, and SPSP-MEO9 film. Data are given as mean ± SD (*n* = 6). Data with different lower case letters indicates significantly different values (*p* < 0.05).

Smart packaging films should possess certain mechanical properties to retain their appearances and to protect food products. TS and EAB are two common indices used to individually compare the stiffness and flexibility of films. As displayed in Figure 4, both the TS and EAB of the films declined after the incorporation of MEO (*p* < 0.05), suggesting that MEO reduced the stiffness and flexibility of films. MEO, due to its hydrophobic nature, reduced the interfacial adhesion between different film components. This was confirmed by the decreased hydrogen bonding system of the films after the incorporation of MEO (Figure 2A). Meanwhile, MEO droplets created several porous microstructures and discontinuities within the film matrix (Figure 1), which reduced the intermolecular interactions of the film components. As a result, the SPSP-MEO films tended to present amorphous state (Figure 2B). Other researchers also documented that the TS and EAB of films containing anthocyanins were reduced by essential oils, which was associated with the disrupted film microstructures by essential oils [10,16,20]. In this study, the results also showed that the TS and EAB of the films gradually decreased with the increase in MEO content (*p* < 0.05). This was because the hydrogen bonding interactions of the films decreased (Figure 2A) but the porous microstructures within the films increased (Figure 1) with the increase in MEO content. Similarly, several researchers also demonstrated that the content of essential oils could affect the mechanical properties of films containing anthocyanins [18,20,24,25,40]. Li et al. [20] observed that the TS and EAB of films containing mulberry anthocyanins continuously declined with increasing lemongrass essential oil content, which was in line with our results. Similar trends were detected by Zhang et al. [18] in chitosan/konjac glucomannan films containing elderberry anthocyanins and different contents of oregano essential oil. Wang et al. [40] and Zabidi et al. [25] found that the EAB of films containing anthocyanins gradually increased with the content of essential oils, which was ascribed to the plasticizing effect of essential oils. However, this phenomenon was not observed in the present study, which might be because the MEO content in the SPSP-MEO films was lower than the essential oil content in the films prepared by Wang et al. [40] and Zabidi et al. [25].

#### 2.2.4. Thermal Properties

**Figure 5:**
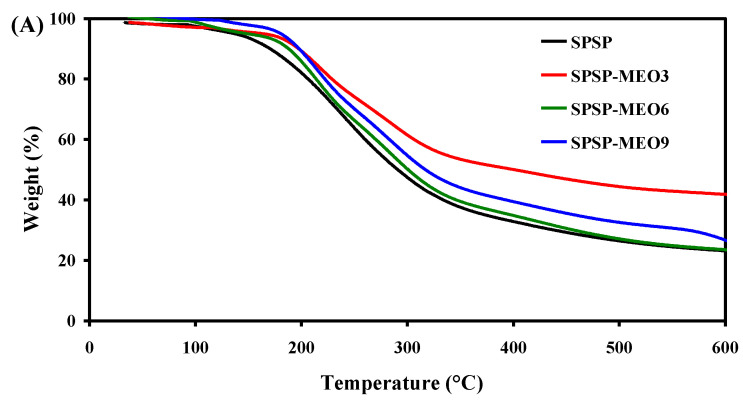

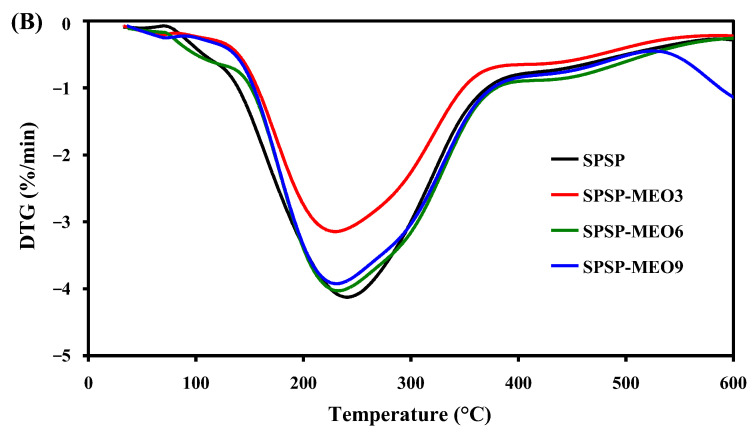
TGA (**A**) and DTG (**B**) curves of SPSP film, SPSP-MEO3 film, SPSP-MEO6 film, and SPSP-MEO9 film.

To test the thermal stability of the smart packaging films, TGA was performed and the results are shown in Figure 5. All films had three degradation stages. The first degradation step, ranging from room temperature to 135 °C, was due to moisture evaporation, while the third degradation step, from 390 °C backward, was caused by the carbonization of the film components [18]. The main degradation process was observed at the second stage, with the most rapid degradation occurring at a temperature of 234–247 °C (Figure 5B). This stage was ascribed to the degradation of high-molecular-weight polymers (e.g., starch, dietary fibers, and sodium alginate) [18,24]. Some low-molecular-weight substances, such as glycerol, anthocyanins, and MEO, were also degraded at this stage [19,26]. Notably, the addition of MEO lowered the temperature of the most rapid degradation of the films, which was because MEO reduced the interfacial adhesion between the film components. As a consequence, the degradation of most film components was faster at this stage. This result was in line with the decreased mechanical properties of the SPSP-MEO films (Figure 4). Nonetheless, the three SPSP-MEO films showed a smaller weight loss than the SPSP film (Figure 5A), indicating that the SPSP-MEO films had elevated thermal stability. Several other researchers also concluded that the thermal stability of films containing anthocyanins was elevated by plant essential oils [18,24,25]. It is worth noting that the SPSP-MEO3 film was the most thermally stable one, with minimum weight loss, which was consistent with its relatively compacter microstructure as compared to the other SPSP-MEO films (Figure 1). The results indicated that the SPSP-MEO films could be used in relatively higher temperature environments.

### 2.3. Functional Properties of Films

#### 2.3.1. Antioxidant Activity

**Figure 6:**
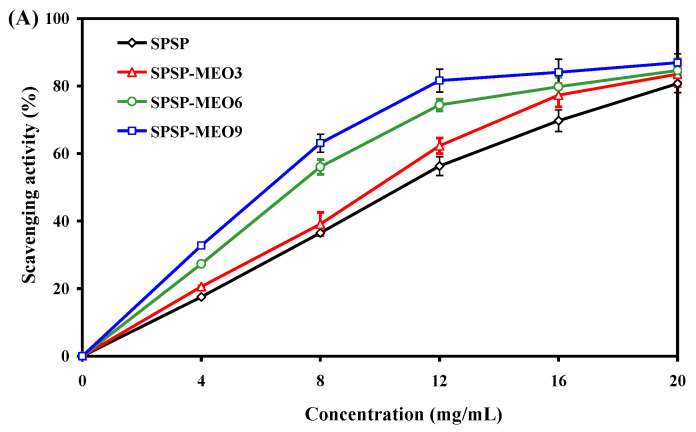

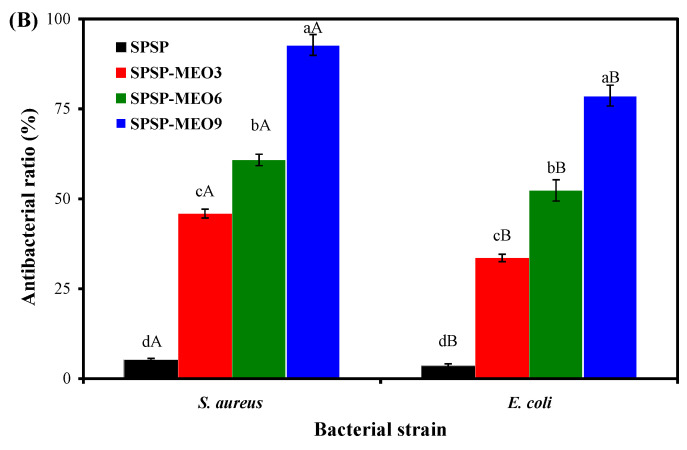
Antioxidant (**A**) and antimicrobial (**B**) activities of SPSP film, SPSP-MEO3 film, SPSP-MEO6 film, and SPSP-MEO9 film. Data are given as mean ± SD (*n* = 3). Different upper case letters indicate the statistically significant difference (*p* < 0.05) within the same film against different bacteria. Different lower case letters indicate the statistically significant difference (*p* < 0.05) among different films against the same bacterium.

The antioxidant activity of the SPSP film, SPSP-MEO3 film, SPSP-MEO6 film, and SPSP-MEO9 film were tested by immersing the films in DPPH solutions. In this way, antioxidants in the films were released into the solutions and could scavenge DPPH radicals. As displayed in Figure 6A, all films had a dose-dependent antioxidant activity. The antioxidant activity of the films progressively increased with increasing sample concentration (*p* < 0.05), attributed to the action of the anthocyanins in purple sweet potato [31]. MEO, due to possessing substantial phenolic and aldehyde components, further elevated the antioxidant activity of the films (*p* < 0.05). The active compounds in MEO, such as thymol, γ-terpinene, α-terpineol, citral, and citronellal, were demonstrated as potent antioxidants with a DPPH radical scavenging ability [38]. In this respect, the antioxidant activity of the SPSP-MEO films increased with increasing MEO content (*p* < 0.05). Several other researchers also detected an enhanced DPPH radical scavenging activity in films containing anthocyanins after essential oils were added [20,21,22,26]. The above results indicated that SPSP-MEO films with a potent antioxidant activity could be used to protect foods from oxidation.

#### 2.3.2. Antimicrobial Activity

The antimicrobial activity of the SPSP film, SPSP-MEO3 film, SPSP-MEO6 film, and SPSP-MEO9 film against Gram-positive *S. aureus* and Gram-negative *E. coli* was evaluated (Figure 6B). The SPSP film showed the lowest antimicrobial activity against the two tested bacteria (*p* < 0.05). A similar result was reported by Chavez-Marquez et al. [16], who found that the films with purple corn cob anthocyanins had no obvious antimicrobial ability. Chen et al. [10] also concluded that cellulose nanofiber films with purple sweet potato anthocyanins had a neglectable antimicrobial potential. MEO significantly increased the antimicrobial activity of the films in this study, with the SPSP-MEO9 film displaying the highest antimicrobial activity (*p* < 0.05). Many researchers also found that films with anthocyanins showed an elevated antimicrobial activity after essential oils were added [10,16,18,20,21,26]. The strong antimicrobial activity of the SPSP-MEO films was attributed to antimicrobial substances in MEO that could disrupt the cytoplasmic membrane of bacteria [38]. It is worth noting that the SPSP-MEO films were more effective in inhibiting the growth of *S. aureus* (*p* < 0.05). Other researchers also found that films with essential oils showed a higher antimicrobial ability against *S. aureus* as compared with *E. coli*, which might be because *E. coli* could metabolize antimicrobial substances and display a certain resistant ability [20]. Yi et al. [38] demonstrated that the inhibition of *S. aureus* was primarily caused by citral, citronellal, decanal, limonene, linalool, octanal, and thymol in MEO, whereas the inhibition of *E. coli* was mainly attributed to citral, citronellal, limonene, linalool, octanal, α-sinensal, and thymol in MEO. The above results indicated that SPSP-MEO films with a potent antimicrobial activity could be used to protect foods from microbial contamination.

#### 2.3.3. Color Change Ability

**Figure 7:**
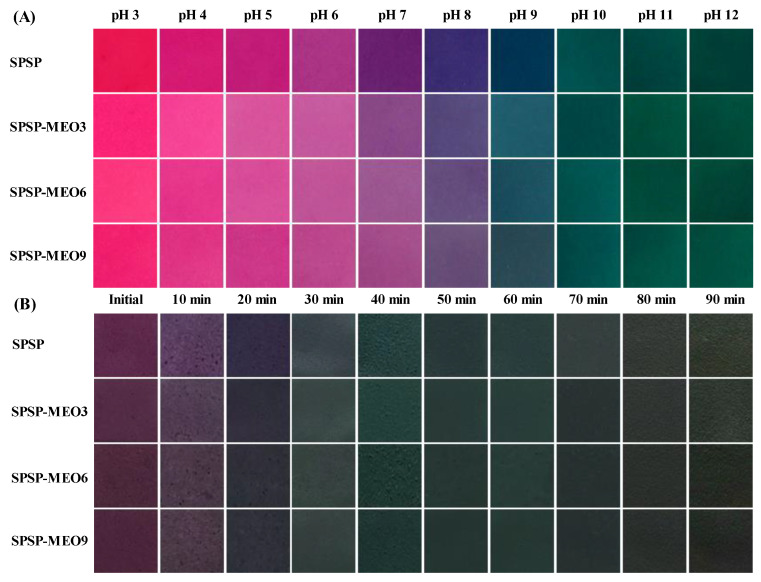
Color change ability of SPSP film, SPSP-MEO3 film, SPSP-MEO6 film, and SPSP-MEO9 film in pH 3–12 solutions (**A**) and ammonia gas (**B**).

The color change ability of the SPSP film, SPSP-MEO3 film, SPSP-MEO6 film, and SPSP-MEO9 film was checked under solutions with different pHs and ammonia gas. As shown in Figure 7, the color of the SPSP film changed from red to purplish red, purple, violet, and green with pH increasing from 3 to 12. In ammonia gas, the purple color of the SPSP film first became shallow and then turned into blue and green when contacting time increased. The color variation was caused by the anthocyanins in purple sweet potato that could change their structures at different acid–base conditions [31]. The three SPSP-MEO films exhibited similar color change profiles to those of the SPSP film, suggesting that MEO did not remarkably alter the color change ability of the films. Previous studies also documented that the color change ability of films with anthocyanins did not change after essential oils were added [10,18,25,40]. Since the food spoilage process is normally accompanied by pH change, pH-sensitive SPSP and SPSP-MEO films could be used to trace the quality of foods through color variation.

### 2.4. Application of Films

**Figure 8:**
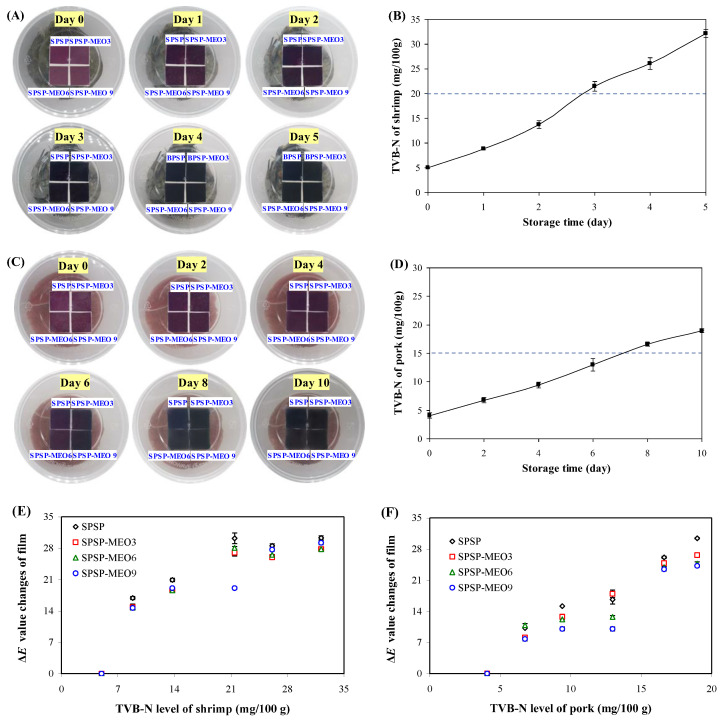
Color changes of SPSP film, SPSP-MEO3 film, SPSP-MEO6 film, and SPSP-MEO9 film for tracing the freshness of shrimp (**A**) and pork (**C**), the TVB-N level of shrimp (**B**) and pork (**D**) during 4 °C storage, and the correlations between the Δ*E* value changes of films and the TVB-N level of shrimp (**E**) and pork (**F**). Data are given as mean ± SD (*n* = 3).

Protein-rich food products, such as shrimp and pork, often have very short shelf lives because of their endogenous decomposition and microbial contamination. Smart packaging films are intended to trace the freshness of protein-rich food products. Here, the SPSP film, SPSP-MEO3 film, SPSP-MEO6 film, and SPSP-MEO9 film were tested for their ability to trace the freshness of shrimp and pork (Figure 8). Obvious color changes (purple → violet) were noted on day 3 for the films used to trace shrimp freshness. As for the films used to trace pork freshness, they displayed color changes (purple → violet) on day 8. It is worth noting that the color changes of smart packaging films should well correspond to the TVB-N changes in protein-rich food products. Figure 8 shows that the TVB-N of shrimp was 21.46 mg/100 g on day 3, approaching the freshness limit of shrimp (20 mg/100 g). In addition, the TVB-N of pork was 16.63 mg/100 g at day 8, slightly higher than the freshness limit of pork (15 mg/100 g). The above results revealed that the films were able to trace the changes in shrimp and pork quality through film color changes. No significant color differences were noted for different films in practical use, which was in line with the previous observation that MEO had little impact on the color change ability of the films (Figure 7). The correlations between the Δ*E* value changes of films and the TVB-N level of shrimp and pork were further analyzed (Figure 8). The results showed that the Δ*E* value changes of the films had higher correlations with the TVB-N level of pork (correlation coefficient of 0.90–0.98), while the Δ*E* value changes of the films had lower correlations with the TVB-N level of shrimp (correlation coefficient of 0.76–0.82). This indicated that the developed films were more effective in tracing the freshness of pork.

## 3. Materials and Methods

### 3.1. Materials, Chemicals, and Microbial Strains

Purple sweet potato (variety Guizi 03) was purchased from Yulin Sweet Potato Corp. (Yulin, China). MEO was obtained from Huashuo Perfume Oil Co., Ltd. (Ji’an, China); it was isolated from fresh mandarin peels using the cold pressing technique. Tween 80, sodium alginate, and 2,2-diphenyl-1-picrylhydrazyl (DPPH) were obtained from Sinopharm Corp. (Shanghai, China). Two bacterial stains of *Staphylococcus aureus* and *Escherichia coli* were stored in our laboratory. Fresh shrimp and pork were acquired from a local supermarket (Yangzhou, China) and transported to the laboratory in boxes with ice.

### 3.2. Steaming Treatment of Purple Sweet Potato

The steaming treatment of purple sweet potato was performed based on the method of Li et al. [31]. Briefly, purple sweet potato was washed with tap water and dried in the shade. Afterwards, purple sweet potato was steamed over a wok which was filled with boiling water and heated at 100 °C for 30 min. The obtained SPSP was cooled to room temperature and peeled, followed by lyophilization and pulverization. The obtained SPSP powder was collected for further use.

### 3.3. Preparation of Smart Packaging Films

Four kinds of smart packaging films with and without different contents of MEO were developed [31]. First, MEO emulsions were prepared by homogenizing MEO and Tween 80 (mass ratio of 4:1) at 10,000 rpm for 4 min. Meanwhile, 6.8 g SPSP powder and 1.36 g sodium alginate were mixed in 170 mL water at 90 °C for 15 min and homogenized at 5000 rpm for 2 min. Afterwards, different contents of MEO emulsions (0, 3%, 6%, and 9%) and glycerol (15%) were added into the SPSP/sodium alginate solution, acting as antimicrobial agent and plasticizer, respectively. The content of MEO and glycerol was calculated based on the total mass (8.16 g) of the SPSP powder and sodium alginate. The obtained film-forming solutions were tested for their pH values (6.4–6.5), degassed, and dried at 30 °C to obtain packaging films. According to the content of MEO emulsions (0, 3%, 6%, and 9%) in the films, the four kinds of films were named SPSP film, SPSP-MEO3 film, SPSP-MEO6 film, and SPSP-MEO9 film. For each type of film, three replicates were prepared and used for subsequent characterization.

### 3.4. Characterization of Smart Packaging Films

#### 3.4.1. Scanning Electron Microscopy (SEM)

Morphological analyses were conducted on Zeiss GeminiSEM 300 (Oberkochen, Baden-Württemberg, Germany) by photographing the surface and cross-section of films at 5 kV. Prior to photographing, films were mounted onto aluminum stubs with double-sided cellophane tape and sputter-coated with a gold layer [17].

#### 3.4.2. Fourier Transforms Infrared (FT-IR) Spectroscopy

FT-IR analyses were conducted on Varian 670-IR Series (Palo Alto, CA, USA) with an attenuated total reflection diamond crystal. The spectra of the films were recorded in the range of 400–4000 cm^−1^. A total of 32 scans were performed for each film at a resolution of 4 cm^−1^ [20].

#### 3.4.3. X-ray Diffraction (XRD)

XRD analyses were conducted by mounting the films on a Bruker D8 Advance diffractometer (Karlsruhe, Baden-Württemberg, Germany) with a Cu/Kα radiation source. Films were scanned in the 2*θ* range of 5–75° at 40 kV and 30 mA [10].

#### 3.4.4. Thickness

Thickness analyses were conducted by trapping films on a micrometer. Ten random measurements were conducted for each film and the average value was recorded as film thickness [27].

#### 3.4.5. Color

The color coordinates (L*, lightness; a*, redness/greenness; and b*, blueness/yellowness) of the films were determined using a 3nh Technology SR-62 colorimeter (Shenzhen, China). Total color difference (Δ*E*) was obtained based on the color coordinates of the films and the control (a white standard plate), according to Roy et al. [41]. Δ*E* was calculated using the following equation:

(1) $$\Delta E=\sqrt{{\left(L^{*}-L_{0}\right)}^{2}+{\left(a^{*}-a_{0}\right)}^{2}+{\left(b^{*}-b_{0}\right)}^{2}}$$

where L*, a*, and b* are the color coordinates of the films, and *L*_0_, *a*_0_, and *b*_0_ are the color coordinates of the control. Three replicates were performed for each type of film.

#### 3.4.6. Light Transmittance

Light transmittance was determined by placing the films in the colorimetric cell of a Lengguang 759S UV/Vis spectrophotometer (Shanghai, China) and scanning the films from 200 to 800 nm [42]. Three replicates were performed for each type of film.

#### 3.4.7. Water Vapor Permeability (WVP)

WVP was measured by sealing the films on permeation cups containing 25 g anhydrous CaCl_2_. The cups were stored at 20 °C inside desiccators, which were maintained at 100% relative humidity (RH) using water. The weight changes of cups were measured for 4 days [43]. Three replicates were performed for each type of film.

#### 3.4.8. Oxygen Permeability (OP)

OP was determined by mounting the films on a Labthink 201 tester (Jinan, Shandong, China) and testing at 23 °C and 50% RH, according to Bi et al. [44]. Three replicates were performed for each type of film.

#### 3.4.9. Mechanical Property

Tensile strength (TS) and elongation at break (EAB) were measured by clamping film samples (6 cm × 1 cm) between two grips (4 cm initial distance) of a Yishite STX200 testing machine (Xiamen, China) and extending the films at 6 cm/min [45]. Six replicates were performed for each type of film.

#### 3.4.10. Thermogravimetric Analysis (TGA)

Thermal stability was tested on a Henven HTG-1 thermogravimetric system (Beijing, China) over the range of 30–600 °C, setting the heating rate as 10 °C/min [12].

#### 3.4.11. Antioxidant Activity

A DPPH assay was conducted by placing the films (1 mg) in 3 mL DPPH methanol solution (100 μmol/L). After 30 min, the films were removed and the rest of the solution was measured for its absorbance at 517 nm [46]. Three replicates were performed for each type of film.

#### 3.4.12. Antimicrobial Activity

The viable cell counting method was conducted to test the antimicrobial activity of the films [47]. Two bacterial strains of *S. aureus* and *E. coli* were grown at 37 °C for 24 h in lysogeny broth medium. The activated strains were diluted with physiological saline to form a bacterial suspension (10^6^ CFU/mL), and then the films (2 × 2 cm^2^) were soaked in 10 mL of bacterial suspension and placed in an incubator at 37 °C for 24 h. After the film specimens was taken out, the bacterial suspension was diluted with physiological saline, spread evenly onto lysogeny broth agar plates, and incubated at 37 °C for 24 h. The colonies on the plate were counted and the antimicrobial ratio of the films was calculated by using bacterial suspensions without films as the control. Three replicates were performed for each type of film.

#### 3.4.13. Color Change Ability

On one hand, the films (2 × 2 cm^2^) were placed for 1 min in pH 3–12 solutions to test their pH sensitivity. On the other hand, the films (2 × 2 cm^2^) were allowed to contact for 90 min with ammonia gas (released from 1 moL/L ammonia water) to test their ammonia sensitivity [31].

### 3.5. Application of Smart Packaging Films in Tracing Food Freshness

Fresh protein-rich food products (shrimp or pork, 150 g each) were stored in a plastic box at 4 °C. Films (2 × 2 cm^2^) were pasted beneath the box lid. The films were allowed to hang over food products and to sense the released volatile ammonia substances. The color of the films was photographed. The total volatile basic nitrogen (TVB-N) level of shrimp and pork was tested in triplicate. Meanwhile, the correlation between the TVB-N level of shrimp/pork and the Δ*E* value changes of the films was analyzed [48].

### 3.6. Statistical Analysis

Three parallel tests were performed for each measurement, except for TS and EAB that were measured six times. The obtained data were analyzed using the statistical software SPSS 19.0 (Chicago, IL, USA) at *p* < 0.05 level.

## 4. Conclusions

Smart packaging films were successfully prepared by incorporating 3%, 6%, and 9% of MEO into an SPSP-based matrix. Different from the SPSP film, the SPSP-MEO films exhibited several internal cavities. The hydrogen bonding interactions and crystalline degree of the SPSP film were reduced by MEO. The incorporation of MEO enhanced the light barrier, water vapor barrier, thermal, antioxidant, and antimicrobial properties of the SPSP film. However, MEO had no significant impact on the color or color change ability of the SPSP film. The content of MEO significantly influenced the cross-sectional morphology, intermolecular interactions, crystallinity, light barrier ability, water vapor barrier ability, oxygen barrier ability, mechanical properties, thermal stability, and antioxidant and antimicrobial activities of the films. The SPSP-MEO films with a color change ability were more effective in tracing the freshness of pork. In future studies, SPSP-MEO films can be also used in active packaging due to their potent antioxidant and antimicrobial activities. It is worth noting that the TS of the SPSP-MEO films was very low; this can be elevated by adding reinforcing agents in the future. Moreover, considering that both the anthocyanins and MEO in the SPSP-MEO films are heat-sensitive substances, it is necessary to seek proper non-thermal sterilization methods for the films.

## Figures and Tables

**Table 1:** Appearance, thickness, and color coordinates of SPSP and SPSP-MEO films.

Film	Appearance	Thickness (mm)	L*	a*	b*	Δ*E*
SPSP	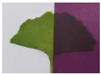	0.139 ± 0.002 ^a^	36.62 ± 0.15 ^d^	34.19 ± 0.47 ^a^	–17.91 ± 0.05 ^d^	63.84 ± 0.05 ^a^
SPSP-MEO3	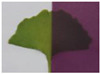	0.140 ± 0.003 ^a^	37.84 ± 0.07 ^c^	27.29 ± 0.59 ^b^	–13.62 ± 0.10 ^c^	58.66 ± 0.33 ^b^
SPSP-MEO6	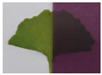	0.140 ± 0.003 ^a^	38.35 ± 0.28 ^b^	24.58 ± 0.28 ^c^	–10.62 ± 0.13 ^b^	56.93 ± 0.32 ^c^
SPSP-MEO9	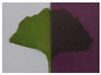	0.141 ± 0.003 ^a^	39.32 ± 0.70 ^a^	23.84 ± 0.25 ^c^	–9.46 ± 0.07 ^a^	55.19 ± 0.19 ^d^

Values are given as mean ± SD (*n* = 10 for film thickness; *n* = 3 for color coordinates). Different letters in the same column indicate significantly different values (*p* < 0.05).

## Data Availability

The data presented in this study are available on request from the corresponding author.
